# Comparative Efficacy of Diethylcarbamazine, Nitazoxanide and Nanocomposite of Nitazoxanide and Silver Nanoparticles on the Dehydrogenases of TCA Cycle in *Setaria cervi*, in Vitro

**Published:** 2018

**Authors:** Sharba KAUSAR, Wajihullah KHAN

**Affiliations:** Section of Parasitology, Dept. of Zoology, Faculty of Life Sciences, Aligarh Muslim University, Aligarh 202002, U.P., India

**Keywords:** Efficacy, Anthelmintics, Nanocomposite, TCA cycle enzymes, *Setaria cervi*

## Abstract

**Background:**

Bovine filariid, *Setaria cervi* may cause serious pathological condition such as cerebrospinal nematodiasis in sheep, goat and horses. Since TCA cycle enzymes have certain biological functions that make them essential for the survival of parasite and therefore, efficacy of diethylcarbamazine (DEC), nitazoxanide (NTZ) and a nanocomposite of nitazoxanide and silver nanoparticles (NTZ+AgNPs) was assessed on succinate, malate and isocitrate dehydrogenases in the microfilariae (mf) and adult *S. cervi* worms.

**Methods:**

This study was conducted in the Department of Zoology, Aligarh Muslim University, Aligarh, India during 2015–2016. Adult and microfilariae of *S. cervi* were incubated in 100 μg/ml of DEC, NTZ, and NTZ+AgNPs for 24 and 6 h, respectively at 37 °C. Succinate, malate and isocitrate dehydrogenases were localized by putting the mf and adult worms in the incubating medium containing their respective substrates at 37 °C for 2 h followed by counterstaining in 2% methylene green for 15 min.

**Results:**

Maximum inhibition of TCA cycle enzymes was observed in both microfilariae and adult worms treated with nanocomposite of NTZ-AgNPs. Ruptured sheath along with nanoparticles sticking to the body surface was noticed in NTZ+AgNPs treated microfilariae.

**Conclusion:**

NTZ+AgNPs proved most effective synergistic combination against TCA cycle enzymes which blocked the isocitrate and malate dehydrogenase almost completely, and succinate dehydrogenase to large extent in both microfilariae as well as adult worms of *S. cervi*. AgNPs ruptured the sheath and allowed the NTZ to attach and penetrate the main body to exert maximum effect on the enzymes.

## Introduction

*Setaria cervi* is an important filariid inhabiting peritoneal cavity of buffaloes causing peritonitis and intestinal occlusion. Adult worms which are generally considered to be non-pathogenic may cause a mild fibrinous peritonitis, but the larval form causes serious condition when they migrate erratically into the central nervous system of unnatural hosts such as sheep, goats and horses (1, 2). Most of the findings related to pharmacology are based on biochemical and physiological aspects of total worm homogenates, as the isolation of different organs and organ systems was not possible. The results thus obtained may not provide possible clue to the biological significance of a particular drug in relation to specific sites. To overcome this difficulty histochemical studies are preferred. Although biochemical studies on TCA cycle enzymes have been studied their localization and physiological role inside the parasite after drug treatment has not been explored extensively.

A few reports are available regarding the effects of anthelmintics on glycolytic and oxidative pathways in the parasites living in the peritoneum, GI tract and tissues of hosts (3–6). Frequent use of the same class of compounds over long periods resulted in resistance among the nematodes of livestock against many anthelmintics (7, 8) and therefore, combination chemotherapy was considered as a powerful strategy to slow it down. Nitazoxanide is a broad-spectrum thiazolide compound possesses anthelmintic, antiprotozoal and antiviral properties (9, 10). Recently nanomedicine has been successfully tried against microorganisms (11, 12). AgNPs formulation is one of them which demonstrated significant effects by inhibiting the proliferation and metabolic activity of promastigotes of *Leishmania* (13).

Keeping above facts in view the present study was conducted to assess the comparative efficacies of DEC, NTZ and nanocomposite of NTZ-AgNPs on the microfilariae and adult worms of *S. cervi*. In vitro effects of these drugs were observed on the microfilariae by scanning-electron microscopy.

## Materials and Methods

This study was conducted during 2015–16. Adult *S. cervi* worms were collected from freshly slaughtered buffaloes and brought to the Parasitology laboratory in the Department of Zoology, Aligarh Muslim University, Aligarh, India. The worms were washed in normal saline and adult females were dissected to recover mf from the gravid segment of the uterus for in vitro study. Effect of DEC, NTZ and NTZ+AgNPs were studied by incubating adult worms and microfilariae in 100μg/ml of these drugs for 24 and 6 h, respectively at 37 °C.

### Localization of Succinate, Malate and Isocitrate dehydrogenases.

Adult worms and microfilariae of *Setaria cervi* were incubated in 100μg/ml of DEC, NTZ and nanocomposite of NTZ and AgNPs for 24 and 6 h, respectively, at 37 °C and then processed for the localization of succinate, malate and isocitrate dehydrogenase. Control and drug-treated microfilariae and adult worms were incubated for 2 h at 37 °C in 18 ml incubating solution (0.2 M Tris buffer, pH 7.4; nitro BT; MgCl2; water) and 2 ml substrate of 2.5 M disodium succinate for the localization of succinate dehydrogenase (SDH), 1 M Malic acid for malate dehydrogenase (MDH) and 1 M trisodium isocitrate for isocitrate dehydrogenase (ICDH). About 2–4 mg coenzyme NAD was added for MDH, and NADP for ICDH enzyme activities. After incubation counterstaining was done by 2% methylene green for 5 min. These sections were then washed and mounted in glycerine and sealed with paraffin. After localization of enzymes, adult worms were dehydrated for 10 min each in ascending ethanol concentration, cleared in xylene and embedded in paraffin wax. Sections were cut at 5 microns, stretched on the slide and dewaxed in xylene. After rehydration in descending grades of alcohol, sections were counterstained in 2% methylene green for 15 min. Sections were dehydrated again, cleared in xylene, mounted in DPX. Slides of both the microfilariae and sections of adult worms were observed under the Nikon Eclipse 600 microscope

### Scanning electron microscopic study

For SEM, microfilariae were incubated in 100μg/ml concentration of DEC, NTZ and NTZ+AgNPs for 6 h. Treated microfilariae were fixed in 2.5% glutaraldehyde in PBS (pH 7.4) for approximately 24 h at room temperature. Fixed mf were washed three times in PBS and stored in it at 4 °C until used. Microfilariae were dehydrated stepwise for 10 min each in ascending grades and kept in 96% ethanol at 4 °C (14). Finally, the microfilariae were dried to critical point, fixed on aluminium stubs and sputter coated with 20 nm gold particles. SEM pictures were taken with a high-resolution scanning electron microscope.

## Results

### Histochemical localization of TCA cycle enzymes (Succinate, Malate and Isocitrate dehydrogenases)

**Fig. 1:**
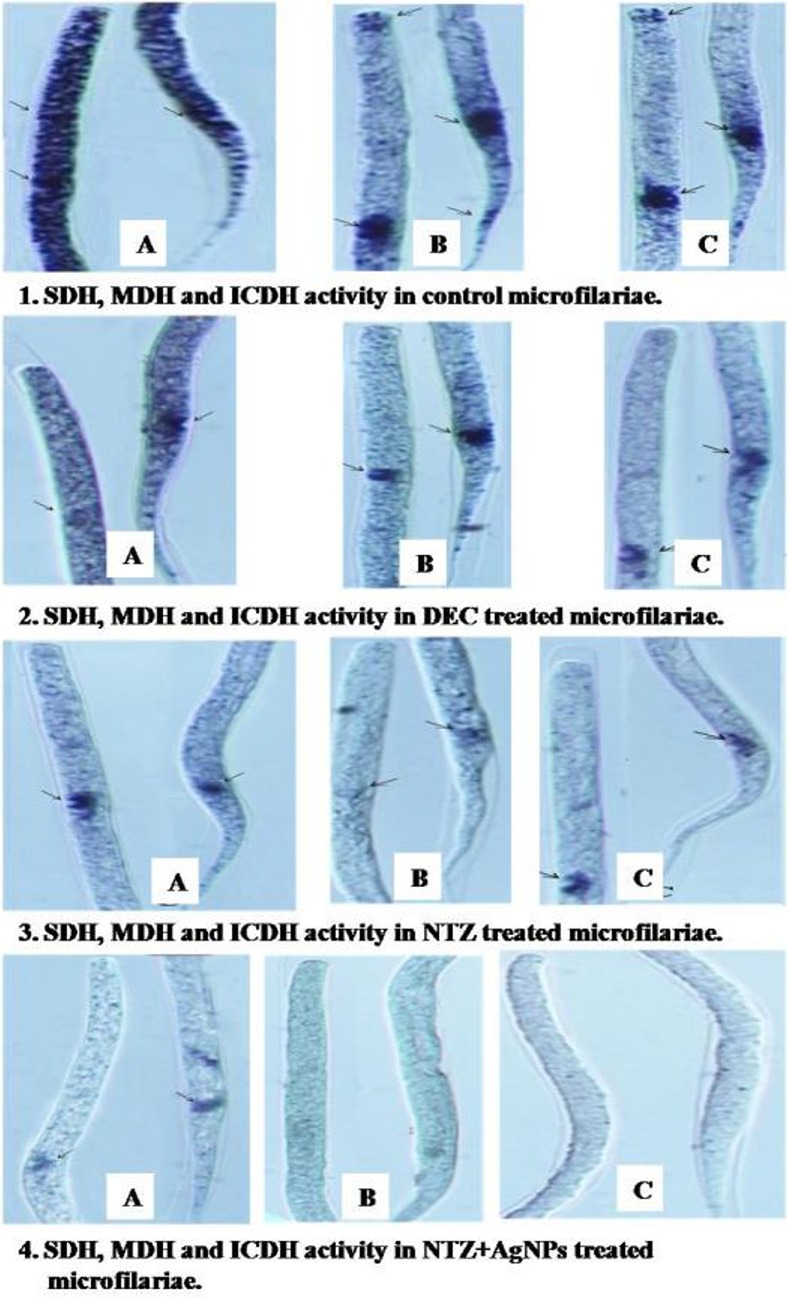
Localization of succinate dehydrogenase, malate dehydrogenase and isocitrate dehydrogenase in control (1), DEC (2), NTZ (3) and NTZ+AgNPs (4) treated microfilariae of *Setaria cervi*

**Fig. 2:**
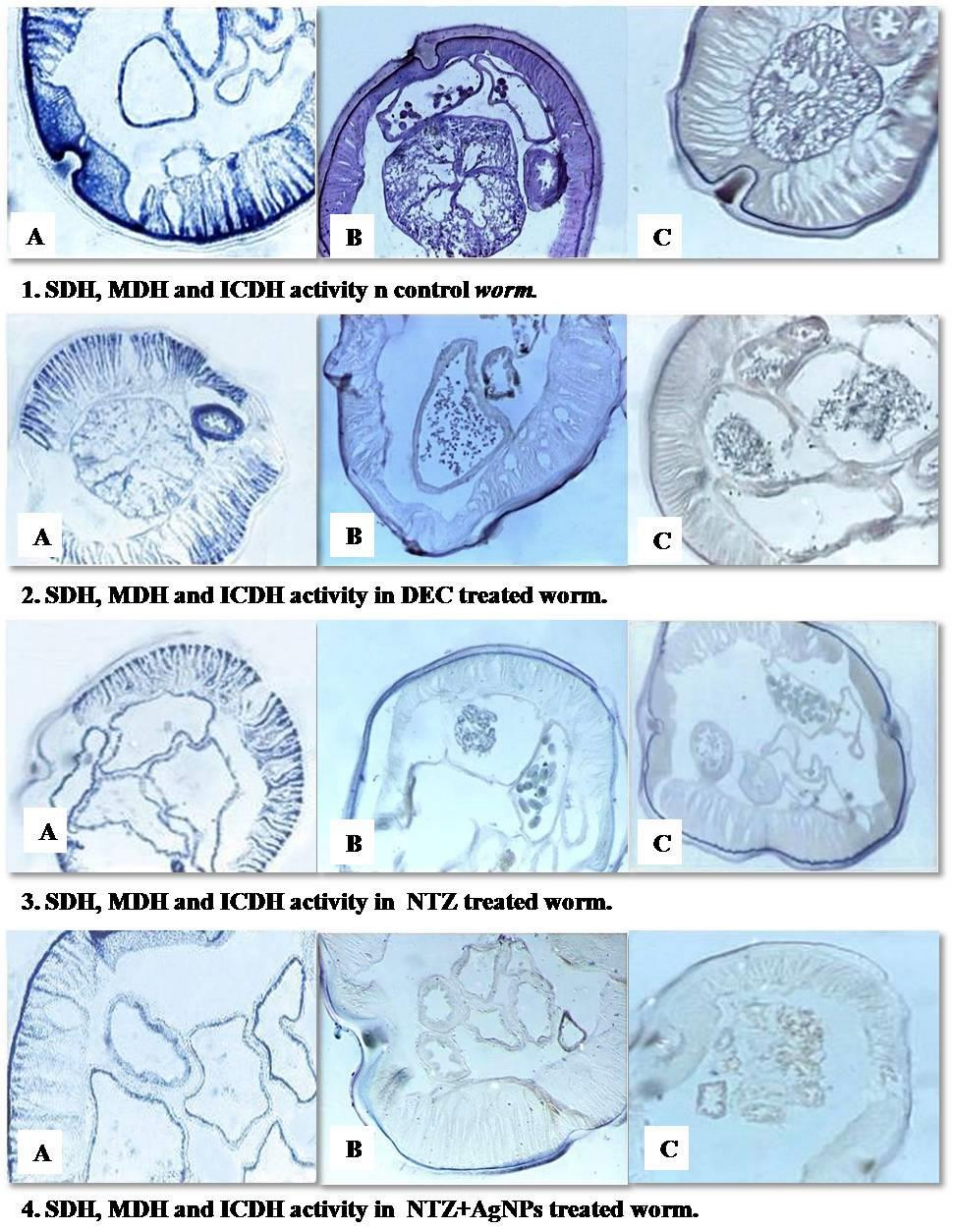
Localization of succinate dehydrogenase, malate dehydrogenase and isocitrate dehydrogenase in control (1), DEC (2), NTZ (3) and NTZ+AgNPs (4) treated *Setaria cervi* worm

Localization of TCA cycle enzymes is shown in Figs. 1, 2. Moderate activity of succinate dehydrogenase was observed throughout the body in DEC, mild in NTZ and negligible in NTZ+AgNPs treated microfilariae compared to control, where intense activity of this enzyme was noticed. SDH activity was moderate to intense in excretory pore and anal pore of microfilariae treated with DEC and NTZ, while it was mild when treated with NTZ+AgNPs. Malate dehydrogenase activity was moderate throughout the body except cephalic cells, excretory pore and anal pore where the reaction was intense in untreated mf, while it was mild on entire body except that of excretory and anal pores where high enzyme activity was observed in the mf treated with DEC. Low MDH activity was observed in anal pore which was very feeble on rest of the body of mf treated with NTZ. In NTZ+AgNPs treated mf, enzyme activity was negligible. Isocitrate dehydrogenase activity was mild throughout the body in normal as well as DEC treated mf, while it was mild and very feeble in the mf treated with the nano-composite of NTZ-AgNPs. In untreated mf, intense activity of isocitrate dehydrogenase was observed in the cephalic cells, excretory and anal pores which was moderate in excretory and anal pores of DEC and NTZ treated mf. However, no enzyme activity was seen in mf treated with NTZ+AgNPs (Fig. 1).

In untreated worms, strong activity of succinate dehydrogenase was observed in hypodermis, fibrillar region of muscle cells, uterine wall and GI tract, while malate dehydrogenase activity was moderate throughout, except the hypodermis which showed intense activity. Isocitrate dehydrogenase activity was also intense in hypodermis while it was moderate in rest of the body parts. In DEC and NTZ treated worms, moderate activity of SDH was observed in musculature, GI tract and uterine epithelium, but was absent from cuticle and hypodermis. NTZ+AgNPs treated worms showed moderate SDH activity in the hypodermis, while rest of the body parts exhibited slight activity. However, no enzyme activity was seen in the cuticle. MDH activity was moderate throughout the body of worm treated with DEC with the exception of cuticle which was devoid of this enzyme. NTZ treated worms exhibited moderate activity of MDH in cuticle and hypodermis, while rest of the body parts showed slight activity. Worms treated with NTZ+AgNPs showed slight activity only in cuticle and hypodermis, while no activity was observed in other parts. DEC treated worms showed moderate activity of ICDH in hypodermis, which was feeble in rest of the body parts. In cuticle, activity of enzyme was totally absent. In NTZ treated worms, slight activity of this enzyme was observed throughout the body except hypodermis which showed moderate activity. NTZ+AgNPs treated worms showed no enzyme activity throughout the body except hypodermis which exhibited slight activity (Fig. 2).

**Fig. 3:**
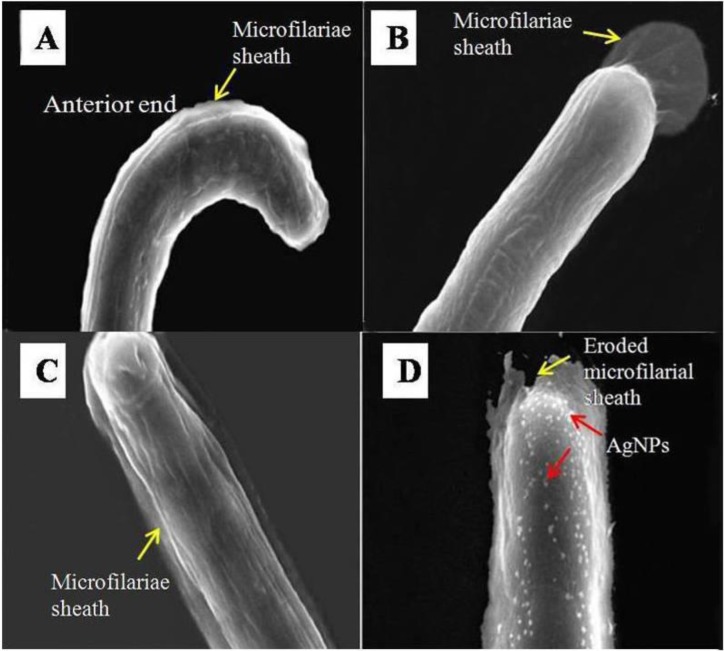
SEM images of microfilariae of *Setaria cervi* treated with Ringer’s solution alone (A) and Ringer’s solution containing 100μg/ml of DEC (B), NTZ (C) and NTZ+AgNPs (D). Yellow arrows point sheath and red arrows point AgNPs

SEM images of the microfilariae treated with 100μg/ml of DEC and NTZ did not show any visible effect on the surface of microfilariae except shrinkage of the sheath when compared with the control. NTZ+AgNPs treated mf showed eroded sheath at few places along with attached nanoparticles (Fig. 3).

## Discussion

Anthelmintics are known to inhibit a variety of enzymes in adult nematodes. NTZ+AgNPs was found to be the most effective inhibitor of succinate, malate and isocitrate dehydrogenase when compared with DEC and NTZ. Localization of these enzymes almost on the entire body of microfilariae and adult worms indicates the presence of PEP-succinate pathway in *S. cervi*. Strong MDH activity on entire body of mf except nerve ring indicated its role in the energy metabolism. Moderate to strong activities of succinate, malate and isocitrate dehydrogenases in the microfilariae and adult worms indicate their role in catalysis of different steps of TCA cycle to generate energy for the worm. Similar intense localization of this enzyme was reported by earlier workers in *S. digitata* and *B. malayi* (15, 16). SDH converts succinate to fumarate, while MDH oxidizes malate to oxaloacetate and vice versa. Intense activity of MDH throughout the body of mf and adult worms suggests faster oxaloacetate reduction and malate formation. Earlier findings indicate that cytosolic MDH when released into the mitochondria for further catabolic processes, affect survival of the parasite adversely (17, 18). Thus, decrease in SDH and MDH activities in treated mf and adult worms point towards the blockage of PEP-succinate pathway and a shift towards homolactate fermentation as earlier reported (19). ICDH enzyme was more pronounced in cephalic cells and anal pore indicating its activity in the parts related to secretion and excretion as earlier observed in *Onchocerca fasciata* (20).

We observed NTZ+AgNPs as most potent inhibitor of TCA cycle enzyme as it slowed down SDH, and completely block the MDH and ICDH activities in treated microfilariae and adult worms. Maximum efficacy of nano-composite of NTZ-AgNPs on both mf and adult worms may be due to the synergistic effect of AgNPs with nitazoxanide in which AgNPs regulate mitochondrial apoptotic pathway and NTZ inhibits the energy metabolism (10, 21).

Moderate SDH and low MDH and ICDH activities in the microfilariae and adult worms after DEC treatment, indicating its inhibitory role in glucose uptake and glycogen synthesis, is in agreement with the earlier findings where similar effects were observed in the mf of *S. cervi* and *Litomosoides carinii* (22, 23). Thus, low levels of SDH, MDH and ICDH in DEC treated microfilariae point towards the retardation of PEP-succinate pathway and a shift towards homolactate fermentation (19).

SEM images in our study did not show any visible effect on the body surface of microfilariae of *S. cervi* except shrinkage after DEC and NTZ treatment. Similar observations were made earlier in the third stage larvae of *Brugia malayi* (24). On contrary to this, changes on the body surface such as wrinkled appearance and sheath loss of the microfilariae of *Wuchereria bancrofti, Litomosoides sigmodontis* and *B. pahangi* were observed in DEC treated worms (25, 26). Removal of sheath exposes the main body of the microfilariae to the antigenic determinants which in turn triggers immune response against the microfilariae afterward and eliminate them slowly. It may, therefore, be presumed that DEC might have different pharmacological mechanism of action against different species of filariids and their developmental stages (27). Effect of NTZ and TZ was also reported in *B. malayi* where these drugs target and cause some structural and functional changes in mitochondria (28). NTZ+AgNPs treated microfilariae of *S. cervi* showed broken sheath alongwith attached nanoparticles on the whole body is in agreement with earlier studies where breaks on the cuticle were observed by AgNPs treatment in *Panagrellus redivivus* and *Caenorhabditis elegans* (29, 30).

## Conclusion

NTZ+AgNPs was the most effective synergistic combination against the TCA cycle enzymes which blocked the ICDH and MDH completely and SDH to large extent in the microfilariae and adult worms of *S. cervi*. AgNPs probably ruptured the sheath which made NTZ accessible to the main body of the microfilariae and produced maximum effect by penetrating through the body wall and acting on the TCA cycle enzymes which play a vital role in the energy metabolism and survival of *S. cervi*.
